# The treatment experience of patients with low back pain during pregnancy and their chiropractors: a qualitative study

**DOI:** 10.1186/2045-709X-20-32

**Published:** 2012-10-09

**Authors:** Shabnam Sadr, Neda Pourkiani-Allah-Abad, Kent Jason Stuber

**Affiliations:** 1 Canadian Memorial Chiropractic College, 6100 Leslie Street, Toronto, Ontario M2H 3J1, Canada

**Keywords:** Pregnancy, Chiropractic, Qualitative, Exercise, Spinal manipulative therapy, Nutrition, Adverse effects

## Abstract

**Background:**

Chiropractors regularly treat pregnant patients for low back pain during their pregnancy. An increasing amount of literature on this topic supports this form of treatment; however the experience of the pregnant patient with low back pain and their chiropractor has not yet been explored. The objective of this study is to explore the experience of chiropractic treatment for pregnant women with low back pain, and their chiropractors.

**Methods:**

This qualitative study employed semi-structured interviews of pregnant patients in their second or third trimester, with low back pain during their pregnancy, and their treating chiropractors in separate interviews. Participants consisted of 11 patients and 12 chiropractors. The interviews consisted of 10 open-ended questions for patients, and eight open-ended questions for chiropractors, asking about their treatment experience or impressions of treating pregnant patients with LBP, respectively. All interviews were audio-recorded, transcribed verbatim, and reviewed independently by the investigators to develop codes, super-codes and themes. Thematic saturation was reached after the eleventh chiropractor and ninth patient interviews. All interviews were analyzed using the qualitative analysis software N-Vivo 9.

**Results:**

Five themes emerged out of the chiropractor and patient interviews. The themes consisted of Treatment and Effectiveness; Chiropractor-Patient Communication; Pregnant Patient Presentation and the Chiropractic Approach to Pregnancy Care; Safety Considerations; and Self-Care.

**Conclusions:**

Chiropractors approach pregnant patients with low back pain from a patient-centered standpoint, and the pregnant patients interviewed in this study who sought chiropractic care appeared to find this approach helpful for managing their back pain symptoms.

## Background

Low back pain (LBP) during pregnancy is reported by approximately 50% to 80% of pregnant women [1-3]. The structural, postural, or hormonal changes that occur during pregnancy, or any combination thereof, may lead to LBP during pregnancy [3]. Treatment options include a range of therapies, such as exercise programs, massage therapy, acupuncture, and chiropractic [4,5]. Chiropractic care may include spinal manipulative therapy (SMT), mobilizations and soft tissue therapy, as well as exercise prescription [3]. Previous studies and systematic reviews of the literature have indicated the relative safety and effectiveness of chiropractic treatment for LBP during pregnancy [3,5-7].

To date, no study has explored the treatment experience of the pregnant population seeking care for their back pain, whether that treatment is from a chiropractor or any other health care provider, nor has any study assessed the experience of chiropractors providing treatment for their pregnant patients with LBP. The qualities of the care provided by the treating chiropractor and that received by the pregnant patient may impact the outcomes of chiropractic care for women with LBP during pregnancy. As such, the objective of this study was to explore the experience of chiropractic treatment for pregnant women who have LBP, as well as their chiropractors in providing care for such patients.

## Methods

This was a qualitative study utilizing semi-structured in-person and telephone interviews conducted in Toronto, Canada. The study was approved by the Canadian Memorial Chiropractic College Research Ethics Board prior to commencement.

### Participants

Participants consisted of 11 patients and 12 chiropractors. The inclusion criteria for pregnant patients in the study consisted of adults (18 years or greater), in their second or third trimester with uncomplicated pregnancies (i.e. absence of pre-eclampsia, gestational diabetes, multiple pregnancies, etc.), who were experiencing LBP during their pregnancy and were seeking treatment for relief of their low back pain symptoms from a chiropractor at the time. Chiropractors included in the study were either male or female adults of any age, who were actively seeing at least one pregnant patient with LBP and agreed to participate in the study both by referring at least one patient and by completing their own semi-structured interview. Signed informed consent was obtained from all participants prior to being interviewed. All transcribed interview records were kept confidential, with only the investigators having access to the information provided. For referencing and analysis purposes a subject identification code was assigned to the each participant based on being a patient (PT) or Doctor of Chiropractic (DC), the numerical order in which they were interviewed for their particular participant category and the date of their interview. For example, the first chiropractor interviewed on August 7, 2011 was coded as: DC01-080711.

### Participant recruitment

Chiropractors were recruited through convenience sampling using chiropractors known to the study team or recommended by other participating chiropractors. The inclusion criteria for patients were explained to the chiropractors who agreed to participate, and they were asked to recruit any of their patients who they determined would meet the inclusion criteria. As such, any pregnant women who met the inclusion criteria were initially asked by their treating chiropractor if they were interested in participating in this study, and were then referred to the investigators to confirm their interest, eligibility for inclusion, and availability for an interview. Most of the chiropractors recruited were practicing in Southern Ontario, while a few were based in Calgary, Alberta.

### Data collection

Semi-structured interviews were carried out in-person for those participants who were available on the CMCC campus clinic or lived within the Greater Toronto Area. Telephone interviews were conducted with those who lived or practiced outside of the Greater Toronto Area. The patient interviews lasted between 15 and 20 minutes and consisted of ten open-ended questions, asking them about their pregnancy and their chiropractic treatment experience for their LBP during the pregnancy. The chiropractor interviews lasted between ten and fifteen minutes and consisted of eight open-ended questions, asking about their experience and impressions of treating pregnant patients with LBP. Two investigators were present at all times for the duration of each interview, and questions were asked by the same investigator for consistency. Question sequencing was flexible, allowing participants to elaborate on their experiences and for questions to be posed at the most appropriate time of a given interview as determined by the investigator. All interviews were conducted between the months of June and November, 2011.

### Data analysis

All of the interviews were audio-recorded using two digital devices, subsequently transcribed verbatim by one of the investigators, and later reviewed independently by the two remaining investigators for completeness and accuracy. Using a grounded theory approach, two investigators independently coded each interview, and all three investigators developed super-codes based on the initial codes, and developed themes from there. Investigator meetings were held where emergent themes, patterns and connections were discussed and reviewed by each investigator to ensure accuracy. During these investigator meetings, it was discussed as to whether or not thematic saturation had been reached. In particular after the eighth chiropractor and eighth patient interviews the three investigators discussed whether or not thematic saturation had been reached and it was felt that it had not yet been met. Subsequently all of the investigators concurred that thematic saturation was reached after the eleventh DC and ninth PT interviews. The remaining interviews allowed for elaboration of the themes. All interviews were imported, organized into themes and analyzed using the qualitative analysis N-Vivo 9 software.

## Results

The 11 included patients were between the ages of 24 and 36 years and the timing of their pregnancies ranged from 17 to 38 weeks. The 12 included chiropractors were in practice between three and 38 years. During analysis five themes emerged from the DC and PT interviews. The themes identified were: Treatment and Effectiveness; Chiropractor-Patient Communication; Pregnant Patient Presentation and the Chiropractic Approach to Pregnancy Care; Safety Considerations; and Self-Care.

### Treatment and effectiveness

Nearly all of the chiropractors indicated that chiropractic treatment was effective in relieving the LBP of pregnant patients. Most of them indicated that they saw positive results in their pregnant patients, for instance: (DC02) “*their symptoms seem to improve with the treatment, and also objectively I find that the joint restrictions improve as well*.” One chiropractor spoke of some of the important outcomes of care for pregnant patients: 

"DC10: “The impact for the pregnant patient is usually profound, as being able to reduce pain and increase function and getting them back into exercise, which is all important for the outcomes of the pregnancy.”"

In terms of treatment procedures, most of the chiropractors indicated employing the Diversified technique, previously reported by 86% of surveyed Canadian chiropractors, [8,9] employing high-velocity, low-amplitude spinal manipulation maneuvers [10], as well as soft tissue therapy and exercise prescription. One particular chiropractor, among the most experienced of those interviewed, said that her treatment plan depended on the patient: 

"DC09: “depends on what the patient comes in with, and it also depends on what time they’re at in their pregnancy, which month. So there are a few variables to consider. We can say essentially Diversified, but again I modify it according to the presenting symptoms and the spinal configuration of the patient.”"

A few of the chiropractors used instrumented techniques for spinal manipulation, such as the Activator® [9,11] or the Integrator^TM^[9] when working on any sensitive areas towards the latter parts of the pregnancy. Two of the chiropractors used the Webster technique [12]: 

"DC05: “The second component of the Webster technique just requires some soft tissue work for the round ligament. I find that works quite well, people respond really well to it.”"

All of the interviewed patients reported that they found chiropractic treatment to be effective and that it helped relieve them of their LBP and associated symptoms. Patients reported that chiropractic treatment had improved their daily living activities and their mobility, while it decreased their overall pain and discomfort:

"PT01: “I can walk longer periods of time. So that’s excellent. I can go standing for four to five hours. Because prior to that I’d be standing 40 minutes to do the dishes and I’d be in agony. even the basic things, like picking things up off the floor, you’d get stuck in that position, I haven’t experienced that yet [in this pregnancy]. Or just sitting for long periods of time, because I do work an office job from home, so I do sit long periods of time. I know for most people [they] have to get up and stretch for a couple minutes, even in doing that I’ve been doing okay.”"

"PT02: “The pain is gone. but I do feel like I have more energy and I can do more activities. The pain doesn’t prevent me anymore.”"

"PT05: “It really allowed me to function. I could barely walk before or stand, the pain was intense, but after I went to the chiropractor. I found I could function day to day, I could walk from the bus stop to work, I could do these things, so that was pretty significant. It’s just helping me function. It’s just teaching me how to pick up my daughter so I don’t hurt myself. To still function, to still play with my daughter and be able to go to the grocery store and do all these things without really hurting myself. As well as it allows me to sleep at night.”"

"PT06: “I found within five days I had greater mobility in terms of twisting and turning. I’m a drama teacher so I’m constantly up on my feet and I need to be able to lean down, jump over, so I need to have a full range of motion, and I found that my range of motion had been very limited and I was getting cranky because I was in pain. I think after the treatment, it was decreased pain and increased sense of mobility, increased range of motion and decreased sense of frustration and grumpiness.”"

"PT11: “that’s the whole reason I go, [for] the pain relief, especially now that I’m a stay-at-home mom, and picking up a 30-pound, 19 month-old all day long. So seeing a chiropractor really helps because it’s not ideal to be lifting a child all day long when you’re in a lot of pain, just the pain relief and the effect that it has on making your daily life easier.”"

### Chiropractor-patient communication

The chiropractors generally emphasized communicating well with their patients as part of providing care and outlining the outcomes to expect. A few of the chiropractors placed great emphasis on patient education about pregnancy, particularly with respect to the changes that are taking place in the patient’s body as they are going through their pregnancy, or the various reasons behind their LBP symptoms. For instance, with respect to doing yoga, DC09 said: “*I encourage that if there are no contraindications, and I give them simple postural advice and things they can do at their workplace*.”

One chiropractor said educating his patients is 50% of what he does, and that it is (DC11) “*a great component initially*”: 

"DC11: “I think the more knowledge they have, the better they are… the woman who is going through the first pregnancy is very scared, hesitant, anxious and wants that kind of knowledge, and wants the practitioner to know what they’re going through and set their mind at ease.”"

Chiropractors were generally open to referring their patients to other professionals if necessary. DC12 said when a patient comes in with a concern “*that is a bit out of my scope*,” he encourages her to contact her midwife or OB-GYN.

One pattern that emerged from the interviews was that communication between chiropractors and patients depended on the knowledge level of both parties. Only a few of the patients seemed to be very knowledgeable about their pregnancy and asked questions or challenged their chiropractors about various techniques or treatments:

"PT06: “I think we sort of have more of a dialogue about what treatment options to pursue, I think I’m more assertive in terms of asking ‘why’ or ‘how come’ or ‘is this a good option’ and ‘what else could we do’.”"

The approach of one chiropractor in particular was shown to help her patients emotionally regarding the changes going on in their bodies. While educating the patient, the chiropractor also eased their mind simply with her communication skills, as explained by her patient:

"PT06: “It’s been emotionally helpful because at the same time I’m getting advice beyond… there’s been explanations of ‘physiologically this is what’s happening to your body’ and ‘this is why your ligaments are pulling’, ‘this is why you’re compensating with the extra weight at the front’, ‘this is why your posture is changing’… and things like that.”"

### Pregnant patient presentation and the chiropractic approach to pregnancy care

Each chiropractor’s approach to treatment depended on their knowledge and experience with pregnant patients. A few of the chiropractors had more preventive maintenance and wellness-based practices where they provided a long-term treatment plan for their pregnant patients. For instance, patients went for regular visits and adjustments, regardless of having any pain or symptoms. One pattern that emerged among the chiropractors, was that most of the pregnant patients they saw and treated were previous “on-going” patients who later became pregnant, and then continued with modified care throughout their pregnancies. In terms of their specific approach to treatment, one chiropractor said:

"DC05: “I definitely take a very holistic approach with my health history and my assessment. I want to get a good sense of what their pregnancy has been like, from the overall standpoint. What type of stressors have been in their world, physical stressors, chemical stressors, the foods that they eat, nutrition is a big piece, activity is a big piece, any mental or emotional concerns or fears going into labor, birth or even pregnancy in general. It can be an emotional time for some women.”"

Another chiropractor (DC06) who frequently treated pregnant women in his practice had a “protocol” that he used with them, and believed that chiropractic care is a great “adjunct” to treating these patients, as part of their larger health care team.

Pregnant patients present differently in terms of their LBP symptoms, its onset, location and duration. The chiropractor’s approach depended on the patient’s presentation, and most of them had a patient-centered approach:This was emphasized by another chiropractor:

"(DC03) “Some will respond differently than others and obviously not every pregnant patient with back pain has exactly the same issues as well. So, we obviously tailor those situations to patients.”"

"DC09: “There are a variety of pregnant patients that come in, some who are ‘die-hard’ chiropractic patients who really don’t care or ask you anything. They just know they are going to feel well, so they just want to be there to continue. Then there are the others who kind of want to try it to see where they’re at, so the first trimester patient in that sense can be particular. The second trimester patient will probably ask more questions because they’ve heard more about chiropractic, so they know it can be beneficial in terms of their delivery especially. they are there for a reason and want to know how often they can come.”"

DC09 further added, “*The bio-psycho-social model is very relevant too. Because they are not all coming to me from nice family units… I’ve got single moms, I’ve got pregnancies that have gone particularly bad… that has a huge impact on the pregnancy as well*.” One chiropractor simply said: (DC12) “*It depends on what trimester they start at and how far along they are and what stresses they have on their body*.”

Many of the patients had received chiropractic treatment prior to their pregnancy. Depending on their symptom presentation and background (in terms of previous pregnancies), some patients received chiropractic treatment when they felt the need, or when they felt their symptoms were flaring up. One patient said: (PT05) “*We don’t really have a treatment plan. It’s more like, ‘when it hurts, come back’. So I’ve just been keeping tabs on how I’m feeling and when it hurts I go back*.”

A few patients received long-term spinal care from their chiropractors who had more of a preventive maintenance and wellness-based approach to therapy, where they received treatment on regular visits for the duration of their pregnancy, even when no symptoms were present, while others were receiving treatment for other conditions and continued on with the care when the low back pain during their pregnancy came on:

"PT03: “I’ve seen chiropractors over the years. I started seeing chiropractors when I was 17, I had low back problems by then and yeah, with the pregnancies it was just getting worse.”"

"PT01: “prior to the pregnancy I used to see the chiropractor for my mid back section and my neck for headaches, so that’s how it all started.”"

Several patients chose chiropractic care for their back pain because they had found results from their first pregnancy:

"PT11: “it worked so well the first time, I’d even come away from the appointment feeling better. It was almost an immediate fix. So because of my positive experience the first time, there was no question that I was going to use chiropractic care for the second pregnancy.”"

Generally, most of the chiropractors were knowledgeable about treating pregnant patients and had either some kind of formal post-graduate training, or had attended seminars or workshops. A few of them said they kept up-to-date with the research on pregnancy, although they admitted, (DC09) “*there is not much*” or (DC12) “*there is not a lot out there*.”

### Safety considerations

All of the treating chiropractors directly stated in their interviews that they believed chiropractic treatment for their pregnant patients was safe, and they had seen no adverse effects. For instance, one chiropractor said: (DC03) “*I primarily do Diversified adjusting in my practice and I consider that to be safe*” This was further emphasized by saying: (DC08) “*in pregnant patients the ligaments are very loose and require very little force to adjust, which I feel makes it much more safe, because you don’t have to do a whole lot to get the response you want.*” With respect to the Webster technique, it was said: (DC05) “*the method of assessment and adjustment is very safe. The patient is very comfortable lying prone on the table with the use of pregnancy pillows”*.

In terms of any contraindications to treatment, most of the chiropractors said that they would not use any electrical modalities on their pregnant patients. One said he would not do much treatment in the first trimester (DC06), whereas two said they wouldn’t start their patient on a new exercise program in the last trimester. One chiropractor said he would not use any SMT on a pregnant patient if he knew that she had a miscarriage before (DC04), or if she had early dilation of the cervix (DC09).

All of the patients directly stated in their interviews that they believed chiropractic treatment was safe and they had not experienced any adverse events after any treatment, which has been reported in previous studies [13]: (PT07) “*I wouldn’t say I’ve ever had any side effects*.” Patients also described their comfort levels changing with particular treatments throughout the pregnancy, while their chiropractors generally modified the treatments to make their patients feel safe and comfortable: 

"PT11: “I’m always very aware of what he’s doing to me and where the position of the baby is. I can’t say I’m 100% comfortable, I know it works, and I’ve read quite a number of articles about it. I haven’t experienced any problems, but it’s always in the back of my mind [safety of my baby], especially in the very beginning, not so much right now, because I’m really big now and it has to be done differently. Now the reservations are there but they’re very small, and I think that’s the case with anything in pregnancy. You just want to make sure you’re doing the right thing for your body and the baby.”"

"PT11: “I was on my back for a couple of the adjustments, and now because I’m so big, I can’t lay too long on my back so comfortably and I can’t lay on my front. He’s given me a cushion but it’s not very comfortable, and so the last adjustment I had was primarily on my side and on my back for a short period of time and nothing on my front.”"

### Self-care

All 12 chiropractors discussed advising exercises or encouraging their patients to follow an exercise program before, during or after their pregnancy. Generally, they emphasized exercise throughout the pregnancy: (DC01) “*I do a lot of exercise therapy with my pregnant patients. It could alleviate some of their aches and pain if they worked out on a regular basis with someone who knows what they’re doing and what exercises to give them*.” Many of the chiropractors had a specific exercise plan or regime that they prescribed to their patients, depending on which stage of pregnancy they were in. For instance: 

"DC03: “What we’re recommending are often core-strengthening exercises, and a lot more women these days are much more aware of getting ahead of the game in terms of fitness training during their pregnancy. If we have the opportunity to work with someone earlier in their pregnancy we’d be working harder to get them moving. Once they’re in the latter half of their pregnancy, really what we’re trying to do is maintaining and making sure we’re not adding anything more than their body can handle at that point and time.”"

Almost all the chiropractors who included exercise in their treatment said that they would not start a new exercise program if the patient was not exercising before pregnancy, and they would not introduce anything new later in the pregnancy: (DC04) “*If there is developing low back pain, I would give them… cat-camel, or basic yoga style stretching*.”; (DC05) “*I walk patients through the type of exercises that I recommend to them. Show them and ask them to do it.*”; “(DC06) *Yoga and Pilates is the only thing that I recommend. I try to keep [their] heart rate low*.”; (DC08) “*I avoid extension exercises, but I still give them some pelvic tilts, of course get them to work on their kegels. Again it depends on the stage of pregnancy and assuming there is no complications or risks*.”; (DC12) “*I’m only suggesting very low impact things, and they all seem to do fine and I haven’t had anyone have a hard time with it*.”

One patient said that her chiropractor did not give her any exercises to do, but generally most of the patients were given some sort of exercise regimen to follow, or were encouraged to do yoga, stretches and/or walking. One patient said: (PT07) “*She’s given me a sheet of stretching exercises that are specifically for pregnancy.”* Patients believed that this active component of their treatment empowered them to take care of themselves when in pain: (PT05) *“he gave [me] some stretches to do… I do them as needed… So when my back is particularly sore but I know I’m not going to the chiropractor for a few days, then I’ll do those stretches and go from there*.”

## Discussion

One of the most common symptoms experienced during pregnancy is low back pain, as over half of the pregnant patient population experience LBP during their pregnancy [1,14]. Chiropractic is the third most common type of Complementary and Alternative Medicine (CAM) treatment sought by pregnant patients [15]. This study investigated the chiropractic treatment experience of pregnant patients with LBP, as well as their chiropractors. The comments of the patients and chiropractors lend some support to previous reports in the literature of positive outcomes of chiropractic care for LBP during pregnancy and provide insight as to how or why those outcomes may be achieved [5,15].

The chiropractors in this study emphasized communication with their patients [16,17] and educated them about pregnancy-related changes that they were experiencing and how the chiropractic treatments could be helpful. Pregnant patients often present differently in terms of their low back pain and symptoms, and the chiropractors in this study employed a patient-centered approach [18], where each patient’s treatment plan was tailored to their specific needs and particular timing in their pregnancy. Knowledge and experience of both patients and their chiropractors played an important role in enhancing the doctor-patient relationship, as well as potentially contributing to a more positive therapeutic outcome.

The chiropractors in this study demonstrated concern regarding patient safety and were vigilant in evaluating for the presence of any contraindications to spinal manipulation. The safety of chiropractic treatments, and specifically spinal manipulation for pregnant patients has been evaluated in a previous survey of chiropractors [6] as well as a critical literature review [7]. These studies indicate that chiropractors feel that their treatments are safe for pregnant patients [6] and report that side effects may be rare, although further research is clearly needed [7].

It has been reported that women who exercise during pregnancy are likely to have more energy, fewer mood swings, better able to manage stress, and get more sleep compared with sedentary pregnant women [19,20]. Exercise prescription appears to be an important component of the treatment program as described by the participants in our study. Most of the chiropractors advised specific stretches or exercises for their patients, depending on how far along they were in their pregnancy. Moderate exercise during pregnancy has been shown to improve overall maternal well-being [19,20]. This was also reflected in the responses of the patients, who indicated that their chiropractor gave them some form of exercise to do at home. Those patients who adhered to their exercise program regularly indicated positive results and improved outcomes in their functioning and activities of daily living.

Future research into chiropractic treatment of pregnant patients with low back pain is warranted and the next step should likely involve a larger scale clinical trial at a higher level of evidence such as a randomized controlled trial [3,5,7]. Any future studies should employ well-defined inclusion and exclusion criteria and evaluate commonly used treatments such as those described by the participants in this study. It will also be necessary to utilize validated outcome measures for pain and disability due to low back pain, and track adverse events, while observing a suitable follow-up period.

### Limitations

As this was a qualitative study that employed convenience sampling as opposed to purposive sampling [21], our sample is not necessarily representative of the experience of all pregnant patients who might have low back pain in their second or third trimester, nor of chiropractors who treat pregnant patients. Since only pregnant patients actively under chiropractic care were included, this study did not consider patient responses prior to receiving chiropractic treatment for their low back pain during pregnancy or after their delivery or conclusion of chiropractic care. Using clinicians to refer patients to the study team may have over-emphasized those patients with a particularly strong relationship with their chiropractors or who strongly value chiropractic care and its effects. Furthermore the clinicians may have only referred those patients that they knew or felt had good outcomes and may be more likely to express satisfaction with care, and thus the voices of those who may not have been satisfied with chiropractic care for low back pain during pregnancy may have been under-represented. These factors could limit the transferability of our findings. However, our data has potential for transferability to a wider population, in this case other chiropractors and their pregnant patients with low back pain, as chiropractors and patients from two different jurisdictions (both Ontario and Alberta) were interviewed and the chiropractors involved had a wide range of practice experience. Furthermore, there was a wide age and gestational period range among the patients interviewed. In future qualitative evaluations of this topic, more purposive sampling of both chiropractors and patients would be beneficial, in particular by seeking out pregnant patients independent of referrals from their chiropractors.

Qualitative research inevitably suffers from issues of bias as the researchers invariably have an effect on the participants and potentially the results of a qualitative study through the concept of reflexivity [21]. However several steps were taken to account for potential bias and reduce the influence of reflexivity as our data was triangulated as we interviewed two groups, pregnant women and their chiropractors, again potentially aiding transferability. The presence of multiple team members at each interview and multiple reviewers to evaluate transcripts and generate codes and themes should further enhance the validity and aid the credibility of our findings. Determining the point of thematic saturation is another particular instance where bias could be introduced and the validity of the findings questioned, particularly if the saturation point was chosen too early, but to reduce this possibility multiple meetings were held among the investigators to help ensure that a suitable and agreed upon thematic saturation point was found and that no new themes emerged in subsequent interviews, which were used to elaborate upon the existing themes.

## Conclusions

Our study was aimed at two subgroups that have not been explored together previously using qualitative research methods, thus it provides new information that can contribute to the current literature on pregnancy and chiropractic treatment. The transferability of the findings of this study may be limited to the group evaluated, particularly owing to possible selection bias as the chiropractors selected patients to refer for interviews with the study team. However, based on the comments of the participating patients and chiropractors, it could be suggested that these particular pregnant women with low-risk or uncomplicated pregnancies, who experienced low back pain, appear to have benefited from chiropractic treatment, including spinal manipulation, soft tissue therapy and exercise therapy. No adverse events were reported by the pregnant patients or their chiropractors in response to spinal manipulation received from their chiropractors. The patients involved reported that they were generally satisfied with the chiropractic care they received during their pregnancy, and had positive outcomes in terms of reduction in their low back pain symptoms, and improved range of motion and overall function. Future research into the effectiveness and safety of chiropractic care for LBP during pregnancy is still needed, potentially including mixed methods research and larger scale clinical trials.

## Competing interests

The authors declare that they have no competing interest.

## Authors’ contributions

SS participated in study design and coordination, data collection and analysis, and helped to draft the manuscript. NPAA participated in study design and coordination, data collection and analysis, and helped to draft the manuscript. KJS conceived the study, participated in its design and coordination, data analysis, and helped to draft the manuscript. “All authors read and approved the final manuscript.”
